# Development and evaluation of a telepharmacy service in primary care for home-living older adults in Northern Sweden’s rural areas: protocol for a single-arm interventional study

**DOI:** 10.1136/bmjopen-2025-110198

**Published:** 2025-11-19

**Authors:** Annica Westberg, Pernilla Andersson, Eva Sönnerstam, Sofia Mattsson, Åsa Holmner, Anette Edin-Liljegren, Maria Gustafsson

**Affiliations:** 1 Department of Medical and Translational Biology, Umeå University, Umeå, Västerbotten County, Sweden; 2 Department of Diagnostics and Intervention, Umeå University, Umeå, Västerbotten County, Sweden; 3 The Centre for Rural Health, Region Västerbotten, Storuman, Västerbotten County, Sweden; 4 Department of Epidemiology and Global Health, Umeå University, Umeå, Västerbotten County, Sweden

**Keywords:** Aged, Medication Review, Pharmacists, Primary Health Care, Telemedicine

## Abstract

**Introduction:**

Medication-related problems (MRPs) are common among older adults. The global population is ageing and there are health-related challenges linked to ageing in rural areas. Home-living rural older adults often face barriers to access healthcare, like long distances to healthcare services and poor continuity of care. Telepharmacy is the remote provision of pharmaceutical care, and telepharmacy could be of particular importance for rural older adults to improve their access to clinical pharmacy services and reduce the incidence of MRPs. The objective of this study is to develop and evaluate a novel telepharmacy service in primary care for home-living older adults in Northern Sweden’s rural areas. The primary objective is to evaluate the effect of the telepharmacy service regarding the identification, classification and resolution of MRPs.

**Methods and analysis:**

This study will be conducted as a single-arm interventional study. A total of 100 people ≥65 years will receive the telepharmacy service for 12 weeks. The key principles of the telepharmacy service are to perform medication interviews and follow-up meetings with study participants, to conduct structured medication reviews, to conduct regular electronic medical record reviews and to have interprofessional collaboration with primary care physicians. All meetings will be conducted through video conferencing via a secure virtual care platform. Identified MRPs will be classified, and the acceptance rate of the pharmacists’ recommendations will be evaluated. The results will be presented with descriptive statistics. As secondary objectives, intra-individual changes in participants’ medication adherence, health-related quality of life and beliefs about medicines will be assessed through self-report questionnaires. Statistical analysis will be conducted using two-sided McNemar’s tests. Semi-structured interviews will also be conducted to explore participants’ and healthcare professionals’ experiences and attitudes towards this telepharmacy service.

**Ethics and dissemination:**

This study has been granted ethical approval by the Swedish Ethical Review Authority (registration number 2022-03819-01 and 2024-08441-02). Participant informed consent is required. The results will be published in peer-reviewed journals and presented at scientific conferences.

**Trial registration number:**

NCT05629936.

**STRENGTHS AND LIMITATIONS OF THIS STUDY**

- Introduces and evaluates a novel telepharmacy service with video-based meetings for home-living older adults in Northern Sweden’s rural areas.
- The feasibility of the study design was qualitatively evaluated through individual interviews prior to finalisation of this study protocol.
- The absence of a control group hinders between-group comparisons.

## Introduction

 Multimorbidity and polypharmacy are common among older adults, and older people are at high risk of medication-related problems (MRPs).^1^,^3^ An MRP is defined as an event or circumstance involving medication therapy that actually or potentially interferes with desired health outcomes.^4^ Unidentified and unresolved MRPs can lead to unplanned hospital admissions^5 6^ and readmissions,^7^ increased healthcare costs^8^ and poor health-related quality of life.^9^

Additional health-related challenges are associated with ageing in rural areas and sparsely populated regions (SPRs). Home-living rural older adults often face several barriers to access healthcare, such as long distances to healthcare services, poor continuity of care and insufficient public transportation services.^10^,^14^ The Swedish Agency for Economic and Regional Growth (Tillväxtverket) classifies rural and urban areas based on geocoded statistics using 1×1 km grid squares.^15^ A grid square is considered dense if it has ≥300 people per km^2^. It is considered rural with <300 people per km^2^. If ≥5000 people are living in a cluster of neighbouring dense grid squares, the area is considered urban. If the total population in the cluster is <5000 the area is still considered rural, even if some grid squares are dense. The population density in Sweden is 25.8 inhabitants per km^2^ (in 2024), which, seen from a European perspective, is considered a relatively sparsely populated country.^16^ Västerbotten County, in Northern Sweden, has 5.1 inhabitants per km^2^. Several municipalities in Västerbotten County have a population density of less than one inhabitant per km^2^, which counts as a SPR according to research.^17^ Furthermore, the population in Västerbotten is ageing. This is a global phenomenon, which means that the whole world is facing this demographic challenge.^18^,^20^ There are several municipalities in Sweden, especially in the inland of Västerbotten County, where almost every third inhabitant is ≥65 years (in 2024).^21^

In recent years, telehealth has emerged as a promising complement to in-person care visits, and as a successful strategy to improve patients’ access to healthcare.^22^ Telehealth is defined as the use of telecommunication technologies to provide healthcare services where patients and providers are separated by distance.^23^ Telepharmacy is a subdivision of telehealth and is defined as the remote provision of pharmaceutical care via telecommunication technologies.^24^

There is a growing body of literature that highlights the use of telepharmacy to support the remote provision of clinical pharmacy services.^25 26^ Different telecommunication technologies have been used, such as video conferencing, telephone-based interventions and web-based applications. For instance, pharmacist-delivered telephone consultations and video conferencing have previously shown promising results regarding the provision of medication optimisation and disease management services in a geriatric primary care setting^27^ and in rural areas in the US.^28 29^ In addition, telepharmacy was also effectively used during the COVID-19 pandemic to perform remote medication reviews and to assess patients’ medication adherence.^26^

To our knowledge, there have been limited attempts to use telepharmacy services in primary care for home-living older adults in Northern Sweden’s rural areas. Telepharmacy could be of particular importance for rural older adults to improve their access to clinical pharmacy services and potentially reduce the incidence of MRPs. The objective of this study is therefore to develop and evaluate a novel telepharmacy service in primary care for home-living older adults in Northern Sweden’s rural areas. This telepharmacy service will be provided through video conferencing, and the primary objective is to evaluate the effect of the service regarding the identification, classification and resolution of MRPs. Secondary objectives are to compare participants’ medication adherence, health-related quality of life and beliefs about medicines through self-report questionnaires, between baseline and the end of the study. In addition, semi-structured interviews will be conducted to collect qualitative data about participants’ and healthcare professionals’ experiences and attitudes towards this telepharmacy service.

## Methods and analysis

This study protocol was developed in accordance with the Standard Protocol Items: Recommendations for Interventional Trials 2025 statement.^30^

### Principal study design

This study will be conducted as a single-arm interventional study. Home-living older adults in Northern Sweden’s rural areas will receive a telepharmacy service in primary care for 12 weeks.

### Study setting

This study will be conducted in collaboration with primary healthcare centres situated in Northern Sweden’s rural areas. A complete list of study sites is available at *clinical.trials.gov* (ID: NCT05629936).

The study will primarily be conducted in collaboration with primary healthcare centres in rural municipalities in Västerbotten County in Northern Sweden, potentially extending to neighbouring counties such as Västernorrland and Norrbotten if needed to gather enough participants. Additional healthcare centres will be invited to participate until the desired sample size of one hundred participants is reached.

The Swedish Agency for Economic and Regional Growth’s^15^ classification of rural municipalities will be used in this study. The agency divides rural municipalities into three subgroups: rural municipalities near urban areas, sparse rural municipalities and very sparse rural municipalities. In rural municipalities near urban areas, >50% of the population lives in rural areas, and most of the population is <45 min away by car to an urban area with at least 50 000 inhabitants. In sparse rural municipalities, >50% of the population lives in rural areas, and most of the population is more than 45 min away by car to an urban area with at least 50 000 inhabitants. In very sparse rural municipalities, the entire population lives in rural areas, and the entire population is more than 90 min away by car to an urban area with at least 50 000 inhabitants.

### Study population and recruitment

#### Eligibility criteria

People will be eligible for inclusion if they are (1) ≥65 years, (2) Living at home (ie, not in a nursing home), (3) Registered at primary healthcare centres in rural municipalities within Västerbotten County (and neighbouring counties if necessary) and (4) Using five or more prescribed medications and/or considered as a potential candidate by the primary care physician (PCP). An example of a potential candidate would be a person with multimorbidity, where a structured medication review could potentially improve patient’s medication-related outcomes. According to national regulations, people ≥75 years with at least five prescribed medications are entitled to receive a medication review once a year in primary care.^31^ In the present study, people aged 65 years or older will be included, as people ≥65 years are defined as older people, according to the statistical office of the European Union (Eurostat).^32^ People will be excluded if they are (1) Unable to communicate, (2) Do not speak Swedish, (3) Have a confirmed major neurocognitive disorder or (4) Receive palliative care.

#### Recruitment of study participants

The PCPs will use their patient lists to identify eligible participants. Eligible patients will be sent an invitation to participate, together with written information about the study, and a consent form (online supplemental material 1 (in Swedish)). They will also receive three different questionnaires about medication adherence,^33^ health-related quality of life^34^ and beliefs about medicines.^35^ All documents will be sent by mail to the patient’s home address. People who wish to participate sign the consent form, answer the questionnaires and return the documents by mail to the primary healthcare centre in a prepaid envelope provided by the research group. Through the consent form, the participant permits access to their contact details and electronic medical records. After receiving the signed consent form, a clinical pharmacist in the research group will contact the participant by phone. The participant will receive oral information about the study, and the clinical pharmacist and the participant will schedule an appointment for a video-based medication interview.

### Sample size

The aim is to include 100 participants. This sample size serves the dual purpose of describing the prevalence of MRPs within the study population with good precision and enabling the statistical evaluation of changes in key outcome measures (eg, self-reported medication adherence, measured dichotomously) from baseline to the end of the study through intra-individual comparisons. No comparison group is included.

A priori power calculation has been performed to size the study with respect to the intra-individual comparison of the proportion of adherent participants. Based on the assumptions of a clinically relevant increase in the proportion of adherent participants from 85% at baseline to 95% at the end of the study (ie, an absolute difference of 10 percentage points), and an expected correlation (phi coefficient) of 0.6 between the two measurement time points, 80 participants are required to achieve 80% statistical power to detect this difference using a two-sided McNemar’s test at a 5% significance level (α=0.05), with continuity correction applied. The assumptions about the proportions of adherent participants are based on results from previous studies on self-reported medication adherence using the Medication Adherence Report Scale (MARS-5) questionnaire.^36 37^

Since the planned inclusion is 100 participants, which exceeds the calculated requirement of 80, the study will have >80% power to identify a difference of this magnitude under the given assumptions. The sample size also accounts for a potential dropout rate of 20%. The sample size of 100 participants is therefore considered well-justified and adequate to meet the study’s descriptive aims and allow for meaningful analyses of changes over time.

### Telepharmacy service

**Figure 1:**
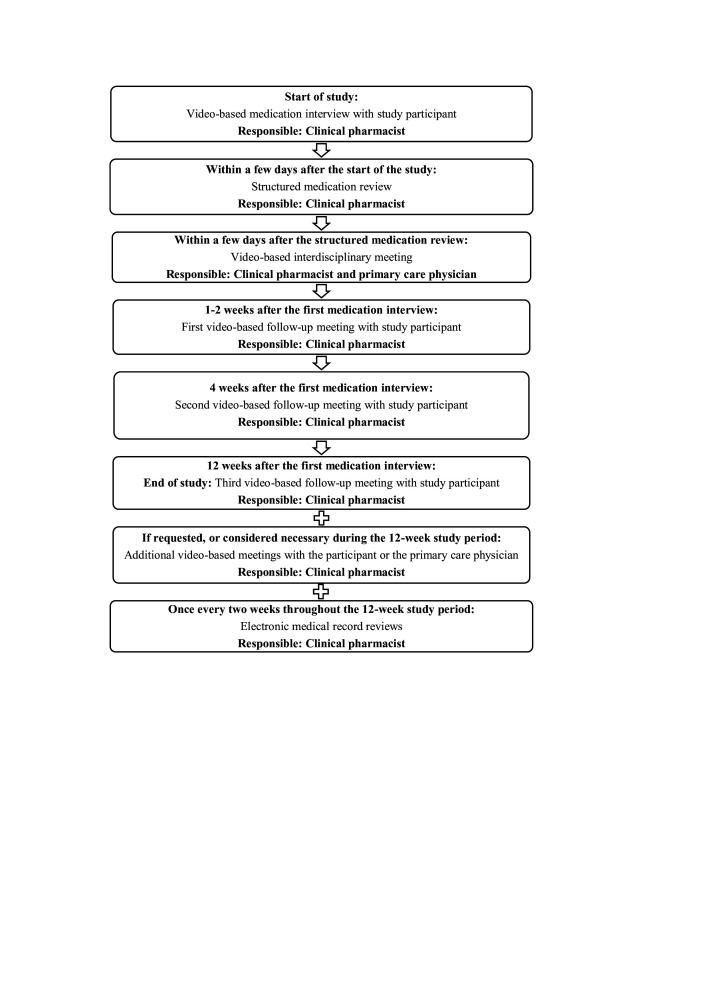
Flowchart of the telepharmacy service.

The key principles of this telepharmacy service (figure 1) are to perform medication interviews and follow-up meetings with study participants, to conduct structured medication reviews, to conduct regular electronic medical record reviews and to have interprofessional collaboration with PCPs. All meetings will be conducted through video conferencing via Visiba Care,^38^ which is a secure virtual care platform. It is used for remote healthcare consultations by several healthcare providers, among others Region Västerbotten. The participants will be able to attend the meetings from home through their own electronic devices such as computers, phones or tablets. The meetings will not be recorded.

All clinical pharmacists performing the intervention will hold a degree of Master of Science in Pharmacy or equivalent qualifications. They should have been previously trained to conduct structured medication reviews or hold relevant education in clinical pharmacy.

This telepharmacy service will be a complementary service to the participants’ regular care. In the case of incidental findings, these findings will be communicated to the participant’s PCP, who either handles the case themselves, or forwards it to the appropriate healthcare unit. Furthermore, the participants will be covered by the ordinary insurance cover for patients within Swedish primary care.

#### Video-based medication interview with study participant

Initially, the clinical pharmacist schedules a meeting for a video-based medication interview with the participant. During the meeting, the clinical pharmacist conducts a medication reconciliation to obtain a complete and accurate list of the participant’s current medications. The pharmacist also asks questions about the participant’s medication management, medication adherence, drug allergies, the use of over-the-counter (OTC) medications, dietary supplements and herbal medicines. The clinical pharmacist can also respond to participants’ medication-related questions. During the meeting, the clinical pharmacist also assesses if the participant experiences symptoms that may be related to their medication therapy, by using the Swedish version of the PHArmacotherapeutical Symptom Evaluation-20 questions (PHASE-20) tool.^39^ PHASE-20 is a validated assessment tool that is frequently used in medication reviews to identify symptoms that may be caused by the patient’s medication therapy. The tool is comprised of 19 symptoms or sets of symptoms that are considered common regarding adverse drug effects in older adults. The tool also contains an open question, if the respondent wishes to address additional symptoms than the pre-formulated ones.

#### Structured medication review and video-based interdisciplinary meeting with the primary care physician

Shortly after the medication interview, the clinical pharmacist conducts a structured medication review (table 1) based on information from the interview and the participant’s electronic medical records. Potential MRPs that cannot be resolved through direct contact with the participant are presented and discussed with the PCP during a pre-scheduled meeting in Visiba Care. During this interdisciplinary meeting, the clinical pharmacist presents recommendations to resolve potential MRPs, and the PCP may accept or reject the pharmacist’s recommendations. The PCP then informs the participant about any changes in their medication therapy and follows up and monitors these changes.

**Table 1:** Structured medication review principles

Medication reconciliation	Identify an accurate list of current medications according to the participant’s statements and electronic medical records
Current health conditions and diseases	Are there valid medical indications for all medications in the medication list? Any untreated conditions?
Overall review of current medication treatments	Check for desired therapeutic effects. Determine if the choice of medications, doses, dosing intervals and treatment durations is appropriate according to current clinical guidelines and recommendations
Side effects, contraindications and inappropriate medications	Check for possible side effects or adverse events. Check for improper drug selections or contraindications, for example, due to certain medical conditions or impaired body functions such as reduced renal or hepatic function
Clinically relevant interactions	Check for drug-drug interactions and food-drug interactions
Medication adherence	Assess whether the participants take their medications as prescribed. Medication non-adherence can be intentional (deciding not to follow the treatment regimen) or unintentional (eg, due to a misunderstanding of treatment instructions or being unable to pay for the medication)
Practical aspects of medication administration	Assess whether the participant has swallowing difficulties or problems with opening medication containers. Are suitable/preferred dosage forms being used? Are medical information and dosing instructions clear and well understood?
Cost-effectiveness	Check for cost-effective use of medications that follow current recommendations. Is the current treatment cost-effective, or are there therapeutically equivalent options available at a lower cost?

#### Video-based follow-up meetings with study participants

Within one to 2 weeks after the initial medication interview, the clinical pharmacist schedules a video-based follow-up meeting with the participant. The purpose of this first follow-up meeting is to check whether the participant has understood and initiated the recommended changes in their medication therapy. The purpose is also to answer questions and to assess if any new problems have emerged regarding the participant’s medication therapy. For some participants, the structured medication review may result in multiple medication-related changes, while other participants will not need any changes in their medication regimen. Two additional follow-up meetings are scheduled and will take place four and twelve weeks after the initial medication interview. The purpose of these meetings is to check on the participant’s progress and experiences of their medication therapy. The clinical pharmacist also asks questions regarding practical aspects of the participant’s medication management, medication adherence and the occurrence of possible side effects. Due to practical reasons, all three follow-up meetings can be re-scheduled to the nearest available date.

#### Additional meetings with the participant or the primary care physician

Additional meetings can be scheduled if requested by the participant or the PCP, or if considered necessary by the clinical pharmacist. For example, the clinical pharmacist may seek additional contacts with the participant or the PCP due to medication-related questions or discovery of additional MRPs.

#### Electronic medical record reviews

The clinical pharmacist conducts electronic medical record reviews once every 2 weeks during the 12-week study period. This activity is conducted to discover abnormal laboratory values, unanswered referrals, to detect whether the participant has had any new changes in their medication regimen, or relevant contacts with healthcare services. Additional MRPs may be discovered, and the electronic medical record reviews also serve as an important basis for the video-based follow-up meetings with the participant.

### Data collection and outcomes

Data will be collected through participants’ electronic medical records, during video-based meetings with participants and PCPs, and through self-report questionnaires.

Participant data needed to perform a structured medication review, such as age, sex, current diseases and medical history, will be extracted from the participants’ electronic medical records (table 2). Additional participant data, such as the use of OTC medications and supplements, medication management issues, as well as symptoms and potential side effects, will be collected orally and documented on data collection forms during the video-based meetings with the participants. PCPs’ acceptance rate of pharmacists’ recommendations will be assessed during the video-based interdisciplinary meetings with the PCPs. Complete data collection forms will be made available on reasonable request.

**Table 2:** Variables and outcome measures

Variables	Description	Source of data	Time interval/timing
Participant characteristics	Age, sex, height, weight, laboratory values, current and previous diseases, current and previous medications or treatments, medication management issues, symptoms and side effects	EMR, VBM-P	12-week study period
MRPs and clinical pharmacists’ recommendations	Identification and classification of MRPs, clinical pharmacists’ recommendations to resolve MRPs, physicians’ acceptance rate of pharmacists’ recommendations	SMR VBIM-PCP VBM-P	12-week study period
Medication adherence	MARS-5	SRQ	Baseline and after 12 weeks
Health-related quality of life	EQ-5D-5L	SRQ	Baseline and after 12 weeks
Beliefs about medicines	BMQ	SRQ	Baseline and after 12 weeks

BMQ, Beliefs about Medicines Questionnaire; EMR, Electronic medical records; EQ-5D-5L, EuroQol-5D-5L questionnaire; MARS-5, Medication Adherence Report Scale; MRPs, Medication-related problems; SMR, Structured medication review; SRQ, Self-report questionnaire; VBIM-PCP, Video-based interdisciplinary meeting with primary care physician; VBM-P, Video-based meeting with participant.

Self-report questionnaires will be used to measure participants’ self-reported medication adherence (MARS-5), health-related quality of life (EuroQol-5D-5L (EQ-5D-5L)) and beliefs about medicines (BMQ) (table 2). The data will be collected when the participants enter the study (baseline) and at the end of the 12 week study period. In accordance with the baseline questionnaires, the follow-up questionnaires will be sent to the participants by mail, after the last follow-up meeting that occurs around 12 weeks after the initial video-based meeting. Incomplete questionnaire responses will be excluded from the analyses.

In addition, for future process evaluation purposes, the clinical pharmacists will document the time required for each activity per participant.

#### Medication-related problems (MRPs)

MRPs are the primary outcome of this study. The MRPs will be classified according to a modified version of the well-known classification system by Cipolle *et al:*^40^ *unnecessary drug therapy, needs additional drug therapy, ineffective/inappropriate drug, dosage too low, dosage too high, adverse drug reaction, non-adherence, drug interactions, monitoring need* and *other*. MRPs will be identified during the entire study period, for example through the structured medication review, by reviewing the participants’ electronic medical records and during the video-based meetings. The clinical pharmacists’ recommendations to resolve MRPs will be classified according to a modified version of the system developed by the French Society of Clinical Pharmacy:^41^ *discontinuation of medication therapy, initiation of medication therapy, dose adjustment, switch medication, optimisation of administration, medication monitoring, information to/discussion with the patient* and *other*. Physicians’ acceptance rate of pharmacists’ recommendations will be classified as *accepted recommendation, rejected recommendation* or *unknown*.

#### Medication adherence

Participants’ self-reported medication adherence will be assessed through the Swedish version of the MARS-5.^33 42^ This validated questionnaire consists of five questions describing non-adherent behaviour that is scored on a five-item Likert scale: 1=always, 2=often, 3=sometimes, 4=rarely and 5=never. The sum score ranges between five and 25 points, where a higher score indicates higher levels of self-reported medication adherence. MARS-5 scores will be dichotomised into adherent/non-adherent. The cut-off score will be >22, in accordance with previous studies.^36 43^

#### Health-related quality of life

Participants’ self-reported health-related quality of life will be assessed through the Swedish version of the EQ-5D-5L.^34 44^ This validated questionnaire consists of two parts, the EQ-5D descriptive system and the EQ visual analogue scale (EQ VAS). The descriptive system is divided into five dimensions with one question for each dimension. The five dimensions are: mobility, self-care, usual activities, pain/discomfort and anxiety/depression. The five-level version has five response levels for each dimension: no problems, slight problems, moderate problems, severe problems and extreme problems/unable to. The second part of the questionnaire consists of a VAS on which the respondents rate their perceived health from 0 (being the worst imaginable health) to 100 (being the best imaginable health). Despite the lack of medication-related questions, the EQ-5D is one of the most frequently used generic instruments for evaluating the impact of pharmacist interventions on health-related quality of life.^45^

#### Beliefs about medicines

Participants’ beliefs about medicines will be assessed through the Swedish version of the Beliefs about Medicines Questionnaire (BMQ).^35^ This validated questionnaire consists of two sections, BMQ-General and BMQ-Specific. BMQ-General assesses the respondents’ beliefs about medicines in general and BMQ-Specific evaluates beliefs about medicines prescribed for the respondents’ personal use. BMQ-General consists of three subscales (*harm, overuse* and *benefit*), while BMQ-Specific consists of two subscales (*necessity* and *concern*). The questionnaire consists of 22 questions that are scored on a five-item Likert scale: 1=strongly disagree, 2=disagree, 3=uncertain, 4=agree and 5=strongly agree.

### Semi-structured interviews

Semi-structured individual interviews will be conducted with participants and healthcare professionals after completion of the telepharmacy service. An interview guide will be used, and the questions will mainly focus on the respondents’ experiences and attitudes towards this telepharmacy service. The interviews will be conducted by a trained interviewer in the research group who is not directly involved in the telepharmacy service.

### Data analysis and statistics

Descriptive statistics, for example, frequencies and proportions, will be used to present MRPs. Due to the single-arm design, no comparative analyses will be performed.

Self-reported medication adherence, beliefs about medicines and health-related quality of life will be compared between baseline and the end of the study using appropriate statistical methods. Regarding self-reported medication adherence, an increase of approximately 10 percentage points is expected. Regarding beliefs about medicines, a moderate increase is expected for positive beliefs (benefit and necessity), while a moderate decrease is expected for negative beliefs (harm, overuse and concern). Regarding health-related quality of life, a moderate positive change is expected. Statistical analyses will also be performed to investigate associations between beliefs about medicines and self-reported medication adherence and various demographic factors such as age, sex and number of medications.

Regarding the semi-structured interviews, the interviews will be audio-recorded, transcribed verbatim and analysed using reflexive thematic analysis.^46 47^

### Pilot testing prior to finalisation of the study protocol

Prior to finalisation of this study protocol, the telepharmacy service was pilot tested to evaluate the feasibility of the study design. The pilot study was conducted at a primary healthcare centre in Västerbotten County, in a municipality with an average population density of less than one inhabitant per km^2^. The study was conducted in collaboration with two PCPs at the healthcare centre. Both PCPs had worked at the healthcare centre for several years, and they were in specialist training to become general practitioners.

The recruitment of study participants was initiated in November 2023. In May 2024, 35 people had received an invitation to participate in the pilot study. Ten people gave their written consent to participate. However, two people withdrew their consent during their first contact with the clinical pharmacist. One person misinterpreted the participant information and the other declined due to the use of digital tools.

#### Qualitative evaluation of the study design

Eight older adults received the telepharmacy service (figure 1). Five of these eight participants were invited to participate in a qualitative evaluation of the telepharmacy service. They received written and oral information about the qualitative evaluation, and everyone gave their oral consent to participate. However, one participant was later excluded from the qualitative evaluation due to repeated failed contact attempts. Accordingly, four participants were interviewed. In addition, both PCPs accepted to participate in the qualitative evaluation.

Digital or phone-mediated semi-structured individual interviews were conducted by an experienced interviewer in the research group (ÅH), who was not directly engaged in the telepharmacy service. An interview guide was developed by the research group and was used to gain insights into the participants’ and the PCPs’ prior experiences of technology, their experiences and attitudes towards the telepharmacy service and the telecommunication technology, and to detect suggestions for improvement. The interviews were audio recorded, transcribed verbatim and thematically analysed using a deductive approach based on the themes of the interview guide. Data transcription was also conducted by ÅH. The qualitative results of the pilot are described below, and the results have also been presented at the Nordic Social Pharmacy Conference held in Glasgow in 2025.^48 49^

#### Prior experiences of technology and perceptions of the telecommunication technology

All participants expressed positive attitudes towards the video-based meetings and the telecommunication technology, despite varying previous experiences and attitudes towards digital tools and technical solutions. It was found to be easy to connect to the virtual care platform, and the telepharmacy interviews were conducted without technical problems. One participant (P4) initially received technical support from next of kin prior to the first video-based medication interview. However, no technical support was needed for the follow-up meetings, since the participant then was familiar with the technology and was able to connect to, and use, the virtual care platform themself. P2 expressed no concerns regarding the video-based meetings, although P2 reflected that physical meetings are preferable. The same participant mentioned that virtual healthcare meetings are probably not appreciated by everyone. Another participant (P1) highlighted that video-based meetings may be preferred over physical meetings, since video-based meetings can be conducted in a self-selected and safe environment, which promotes feelings of safety.

(…) It’s… it’s almost easier to talk like this (through a video-based meeting). (…) No, but it’ s like… it’s like if you and I had met in person. If you’d been sitting here on the other side of the table… it would’ve been more difficult. Because now I’m just sitting here (at home), just got up… (laughs)… a bit tired (laughs). But… but… I feel safer this way, I think. (P1)

The PCPs also expressed positive attitudes towards the telecommunication technology, and the video-based meetings were conducted without technical problems. Both PCPs had previous experience of providing healthcare services via telehealth. One of the PCPs (PCP1) reported that today’s digital tools operate so well that a digital meeting between a few people is almost equivalent to a physical meeting. Both PCPs highlighted the potential of virtual care to improve work flexibility and increase the availability of healthcare services.

#### Perceptions of the telepharmacy service

All participants reported positive experiences regarding the medication interviews. They were comfortable discussing their medications with the clinical pharmacist. The meetings were perceived as well-structured, and the content was relevant. Participant P3 said: ‘It was really helpful to go through everything (the medications). And we also went through my supplements, like vitamins and things like that’. None of the participants reported feeling stressed during the meetings. Two participants (P2 and P4) mentioned stress as a barrier to effective communication as it may cause misunderstandings and forgetfulness.

Well, I have to say… speaking freely from the heart, I thought they (the video-based medication interviews) were really good because I got answers to many of my questions. So, it was a positive experience, and the person (the clinical pharmacist) I got to talk to was really kind. And I didn’t feel… I mean, I wasn’t terrified about talking to her, because she was easygoing. So, I only have good… things to say about her. And the setup itself was also… well, I got answers to a lot of questions. (P4)

The PCPs stated that it was rewarding and beneficial to conduct structured medication reviews with the clinical pharmacist. They both expressed positive attitudes towards structured medication reviews in general, and they were familiar with collaborating with a clinical pharmacist in inpatient care. The project did not take up too much of the PCPs’ time, however, one of the PCPs (PCP2) stated that resources need to be allocated to be able to implement this type of service in ordinary care. Both PCPs recognised the pharmacist as an essential member of the healthcare team that can help to optimise medication use and therapeutic outcomes, for example by detecting and managing drug interactions. Improved continuity, efficiency and quality of care were highlighted as possible long-term effects of this service.

It’s easy to connect (to the video-based meetings), I think it’s been good to do the medication reviews. And it’s been rewarding. Uh… most of them have actually led to some changes along the way, some follow-ups and things like that, so… it’s felt really positive to do them (the video-based interdisciplinary meetings with the clinical pharmacist) (PCP2).

(…) But I don’t think this (the telepharmacy service) has taken up an unreasonable amount of time. I actually think it’s quite reasonable, because it’s actually part of what we should be doing anyway. I think it’s actually become more efficient, because I get this fantastic support in going through… the medication lists. So, in that sense, the time it takes I get back many times over in terms of the quality of care I can provide. (PCP1)

#### Improvement suggestions

All participants were satisfied with the telepharmacy service and had no concrete suggestions for improvement. One participant (P2) briefly reflected on generic substitution and the confusion errors that can occur when equivalent medications have different names.

(…) I was really satisfied after each video-based meeting. I—I got to speak freely from the heart, so to speak, and she (the clinical pharmacist) answered as best she could, and I got help when she had spoken to the primary care physician. So, I—I thought it was all really good, so to speak. I can’t say how it could be made better, because…, I got answers to everything I… needed to know. (P4)

The PCPs identified three potential areas of improvement: altered eligibility criteria, laboratory testing prior to the medication review and tripartite meetings between participant, pharmacist and PCP.

One of the PCPs (PCP1) reflected on altered eligibility criteria, since several people didn’t respond to the invitation or declined to participate.

Collecting relevant laboratory tests prior to the medication review was suggested as an additional step in this telepharmacy service. In the pilot study, it was noticed during some of the medication reviews that relevant laboratory tests were outdated or missing. Both PCPs stated that recently collected laboratory tests are valuable information to have access to during the medication review to support therapeutic decision-making.

One of the PCPs (PCP1) mentioned that tripartite meetings may promote effective communication and prevent errors, since the participant can receive consistent and individually tailored information. However, it was also argued that it would be very difficult to schedule for tripartite meetings in ordinary care.

#### Pilot study conclusions

The results of the pilot study indicated that the study design was feasible and acceptable. Participants and PCPs expressed positive attitudes towards the telepharmacy service, and no amendments in the study design were made prior to finalisation of this study protocol.

The eligibility criteria remained unchanged, since the aim of this study is to develop and evaluate this service among home-living older adults. Depending on the results of the main study, it could be interesting to investigate the effects of this telepharmacy service among other participant groups in the future.

Laboratory testing prior to the medication review was not incorporated as an additional step in this telepharmacy service. This measure was deemed unnecessary, since it will be detected during the telepharmacy service if relevant laboratory tests are outdated or missing. In such cases, relevant laboratory tests will be ordered and evaluated.

Due to practical difficulties, tripartite meetings were not incorporated in the final study design. Interdisciplinary pharmacist-PCP collaboration is therefore essential to ensure that the participant will be provided with consistent and personalised information.

### Data management

Data will be pseudonymised and stored on hard copies or on a secure file sharing platform. The hard copies will be organised in separate folders for each participant and will be stored in a locked filing cabinet. The filing cabinet is in a locked office at the first author’s university department that is inaccessible to unauthorised individuals. The code key will be stored separately from the source data in another locked filing cabinet. Only the research group will have access to the file sharing platform, the filing cabinets and the final study dataset. In the main study, a confidentiality agreement will be established with an external party who will transcribe the data from the semi-structured interviews.

### Patient and public involvement

Participants in the pilot study were involved in the development of the final study design. Patients and/or the public were not involved in the formulation of the research questions or in the development of the outcome measures. They will not be involved in the dissemination of the study results.

### Ethics and dissemination

Ethical approval has been granted by the Swedish Ethical Review Authority (registration number 2022-03819-01 and 2024-08441-02). In the event of substantial amendments to the study design, the amendments will be reported to the ethics committee for approval prior to being implemented. Participant informed consent is required and all participants in the telepharmacy service will provide written informed consent (online supplemental material 1 (in Swedish)). All individuals who also participate in the semi-structured interviews will provide oral informed consent, and this will be collected during the interview sessions. Participation is voluntary, and the participants have the right to withdraw their consents at any time. In the case of withdrawal from study participation, the service will be stopped, and no additional data will be collected.

The study is registered in the clinical trial registry ClinicalTrials.gov (NCT05629936). The study results will be published in peer-reviewed journals and presented at national and international conferences. The results will be presented at group level, and it will not be possible to relate data to any specific individual. Individual participant data will not be shared or publicly available due to privacy and ethical restrictions.

## Discussion

This protocol describes a telepharmacy service in primary care in Northern Sweden’s rural areas, a novel intervention that has not been carried out in this setting before. Development and introduction of telepharmacy services can hopefully help tackle the unequal access to healthcare services that can exist between urban and rural areas. This study aims to provide insights regarding the prevalence of MRPs within this population of home-living older adults. A collaborative approach will be used to identify and resolve potential MRPs, with clinical pharmacists contributing as part of the healthcare team. The findings may provide additional support for continued implementation of clinical pharmacy services within Swedish primary care.

Despite its strengths, this study is subject to some limitations. The absence of a control group hinders between-group comparisons. The single-arm design is still considered a suitable choice for obtaining initial insights into our telepharmacy service. Depending on the results of this study, it may be warranted to conduct robust and high-quality studies in the future to verify the initial findings. Insights from the pilot study suggest that this project may face difficulties in participant recruitment. However, through collaboration with several healthcare centres, the desired sample size is expected to be reached. The reason for non-participation is unclear, and it cannot be ruled out that those who participate differ from those who decline. Lastly, self-report questionnaires will be used to detect intra-individual changes in participants’ medication adherence, health-related quality of life and beliefs about medicines. It is important to acknowledge the potential issues with response bias and that medication adherence, health-related quality of life and beliefs about medicines are all multifaceted phenomena where several factors may contribute to the observed effects.

## Supplementary material

10.1136/bmjopen-2025-110198 online supplemental file 1
