# Supplementary material for: Development and evaluation of a telepharmacy service in primary care for home-living older adults in Northern Sweden’s rural areas: protocol for a single-arm interventional study
Source: BMJ Open. 2025 Nov 19;15(11):e110198. doi: 10.1136/bmjopen-2025-110198 (PMC12636900; doi:10.1136/bmjopen-2025-110198)
Supplement: online supplemental file 1 [file bmjopen-15-11-s001.docx]

# Samtycke till att delta i projektet

Jag har fått muntlig och/eller skriftlig information om studien och har haft möjlighet att ställa frågor. Jag får behålla den skriftliga informationen.

- Jag samtycker till att delta i projektet *Förbättrad läkemedelsbehandling och ökad patientmedverkan genom en digital tvärvetenskaplig modell inom primärvården i glesbygden i norra Sverige*
- Jag samtycker till att apotekaren fortlöpande tar ut uppgifter från datajournalen under studietiden

| Plats och datum | Underskrift |
| --- | --- |
|  |  |
|  | Namnförtydligande |
|  |  |
