# Supplementary material for: Development and evaluation of a telepharmacy service in primary care for home-living older adults in Northern Sweden’s rural areas: protocol for a single-arm interventional study
Source: BMJ Open. 2025 Nov 19;15(11):e110198. doi: 10.1136/bmjopen-2025-110198 (PMC12636900; doi:10.1136/bmjopen-2025-110198)
Supplement: Supplementary file 2 [file bmjopen-2025-110198.reviewer_comments.pdf]

PEER REVIEW HISTORY

BMJ Open publishes all reviews undertaken for accepted manuscripts. Reviewers are asked to complete a checklist review form and are provided with free text boxes to elaborate on their assessment. These free text comments are reproduced below.

ARTICLE DETAILS

Title (Provisional)

Development and evaluation of a telepharmacy service in primary care for home-living older adults in Northern Sweden's rural areas: protocol for a single-arm interventional study

Authors

Westberg, Annica; Andersson, Pernilla; Sönnerstam, Eva; Mattsson, Sofia; Holmner, Åsa; Edin-Liljegren, Anette; Gustafsson, Maria

---

VERSION 1 - REVIEW

**Reviewer**	**1**
**Name**	**Castro, Cynthia**
**Affiliation**	**Modern Health, Inc.**
**Date**	**17-Oct-2025**
**COI**	**None**

This is a nicely-written and thorough protocol manuscript. The study purpose, design and methods, are well described, and the methods are sufficient to test the objectives. There are a few areas for improvement:

1. A more thorough Analysis Plan is needed to describe the specific outcomes and how they map to the study aims. The investigators should describe the expected direction of the outcomes, the anticipated magnitude, as well as any mitigating factors/covariates they may add to the equation.

2. The manuscript body is lacking a Limitations section. While one limitation is mentioned in the Abstract, the lessons from the Pilot Study suggest there will be recruitment/retention challenges; these could be included.

3. A forward-looking Conclusion section would be helpful; what do the investigators anticipate they will learn, and how will this inform future research and clinical pharmacy practice? These thoughts help the reading audience grasp the impact and expected value of the work to the scientific and clinical practice communities.

VERSION 1 - AUTHOR RESPONSE

Reviewer: 1
Dr. Cynthia Castro, Modern Health, Inc.
Comments to the Author:
This is a nicely-written and thorough protocol manuscript. The study purpose, design and methods, are well described, and the methods are sufficient to test the objectives. There are a few areas for improvement:

1. A more thorough Analysis Plan is needed to describe the specific outcomes and how they map to the study aims. The investigators should describe the expected direction of the outcomes, the anticipated magnitude, as well as any mitigating factors/covariates they may add to the equation.

Thank you for your comment. We have added a section titled "Data analysis and Statistics", where we describe the different outcome measures according to the suggestions above. In the "DISCUSSION" section, we have included limitations related to the use of self-report questionnaires. In addition, we have clarified the study's secondary objectives to better describe what we intend to investigate.

2. The manuscript body is lacking a Limitations section. While one limitation is mentioned in the Abstract, the lessons from the Pilot Study suggest there will be recruitment/retention challenges; these could be included.

Thank you for your suggestion. We have added a section on limitations in the "DISCUSSION" section (as suggested by the editor). In this section, we address limitations of the study, i.e., the absence of a control group, potential recruitment challenges, and potential limitations associated with the use of self-report questionnaires.

3. A forward-looking Conclusion section would be helpful; what do the investigators anticipate they will learn, and how will this inform future research and clinical pharmacy practice? These thoughts help the reading audience grasp the impact and expected value of the work to the scientific and clinical practice communities.

Thank you for your suggestion. We agree that a forward-looking section would be helpful and that such reflections may help the reading audience to understand the potential value of this work. We have therefore added a section about this in "DISCUSSION" (as suggested by the editor).

*** ***

Reviewer: 1
If you have selected 'Yes' above, please provide details of any competing interests.: Not applicable.
